# Evaluation of Prediction Models for Decision-Making: Beyond Calibration and Discrimination

**DOI:** 10.1371/journal.pmed.1001491

**Published:** 2013-07-30

**Authors:** Lars Holmberg, Andrew Vickers

**Affiliations:** 1 Regional Cancer Center Uppsala Örebro, Uppsala University Hospital, Uppsala, Sweden; 2 Department of Surgical Sciences, Uppsala University, Uppsala, Sweden; 3 Division of Cancer Studies, School of Medicine, King's College London, London, United Kingdom; 4 Department of Epidemiology and Biostatistics, Memorial Sloan-Kettering Cancer Center, New York, New York, United States of America

## Abstract

Lars Holmberg and Andrew Vickers discuss the importance of ensuring prediction models lead to better decision making in light of new research into breast, endometrial, and ovarian cancer risk by Ruth Pfeiffer and colleagues.

*Please see later in the article for the Editors' Summary*

**Linked Research Article**

This Perspective discusses the following new study published in *PLOS Medicine*:

Pfeiffer RM, Park Y, Kreimer AR, Lacey JV Jr, Pee D, et al. (2013) Risk Prediction for Breast, Endometrial, and Ovarian Cancer in White Women Aged 50 Years or Older: Derivation and Validation from Population-Based Cohort Studies. PLoS Med 10(7): e1001492. doi:10.1371/journal.pmed.1001492

Ruth Pfeiffer and colleagues describe models to calculate absolute risks for breast, endometrial, and ovarian cancers for white, non-hispanic women over 50 years old using easily obtainable risk factors.

In this week's issue of *PLOS Medicine*, Ruth Pfeiffer and colleagues present risk prediction models for breast, endometrial, and ovarian cancer [1]. Improvement of existing models and a new model for endometrial cancer can, as the authors say, be useful for several purposes. However, the paper also raises issues about the challenges of model improvement, interpretation, and application to public health and to clinical decision-making.

Ruth Pfeiffer and colleagues present models for absolute risks and thereby avoid the common mistake of proclaiming a substantial relative risk as clinically relevant without considering the background risk. For example, a relative risk of 3.0 corresponding to a risk increase from 1% to 3% may have quite different implications than an increase from 10% to 30% [2].

The key claim of the paper is that the models “may assist in clinical decision-making.” While the examples in the paper predominately concern prevention, rather than what many readers would intuitively think of as *clinical* decision-making—situations such as primary treatment of early prostate cancer or choice of adjuvant chemotherapy for early breast cancer—the emphasis on decision-making is laudable. What we want from models is that they help us make better decisions, leading to better outcomes for our patients. This raises the question of how to evaluate whether a model does indeed improve decision-making.

As the authors state, good calibration is essential for good decision-making. A model is well calibrated if, for every 100 individuals given a risk of *x*%, close to *x* will indeed have the event of interest. Calibration concerns average risk in a population and a well-calibrated model may assist in prevention decisions, but a miscalibrated model may lead to situations where an individual at high risk is assigned a low predicted probability, and thus forgoes effective preventive intervention. However, calibration is necessary but not sufficient for clinical utility, as the example of mammography screening shows: breast cancer risk prediction models are rarely used to determine eligibility for screening, which is instead based predominately on age, because very large differences in risk between women would be needed to justify separating women into higher versus lower intensities of mammography.

The statistical measure of how well a model separates risk is known as discrimination. But traditional analyses of risk factors are, on their own, not well suited to discriminate prognostic groups in a way that is useful for clinical decision-making [3],[4]. Discrimination is often described in terms of the area under the receiver operating characteristic curve (AUC), taken from the receiver operating characteristics. The AUC is often a useful first step in evaluating a model or in comparing two diagnostic or prognostic models against each other. But like calibration, the AUC value is insufficient to demonstrate that a model would improve decision-making [5]. The calculation of AUC assumes that sensitivity is of equal value to specificity, whereas typically the consequences of a false negative (such as a missed cancer) are dramatically different from those of a false positive (such as an unnecessary biopsy). One example is a classifier of aggressive prostate cancer associated with a clearly elevated relative risk of lethal cancer that has an AUC statistically significantly over 0.5, but that still has an unacceptable rate of false negatives that could imply missed treatment opportunities for the ranges where it is reasonable to use [6].

The paper by Pfeiffer and colleagues raises the critical issue of how we should determine the clinical utility of a model, whether it changes decisions, and whether those decisions are good ones. This is an issue that touches a variety of different areas in medical prediction, including comparisons of models and the value of novel molecular markers. Recent years have seen numerous methodological developments, going above and beyond a clear recommendation that clinical utility should be formally assessed [7] to actual statistical techniques for doing so [8],[9]. One of us (A. V.) developed a method for evaluating prediction models called decision curve analysis [10], a straightforward technique with readily available software (http://www.decisioncurveanalysis.org).

Thus, there are now quantitative techniques available that can determine whether a model does more good than harm given reasonable assumptions about the consequences of false negatives compared to false positives. This takes us substantially further than the (unsolved) debate about whether model evaluation should prioritize calibration or discrimination [5],[11]–[14]. Use of novel decision analytic techniques can also avoid the sort of problems raised by statements such as “[w]ell-calibrated risk models, even those with modest discriminatory accuracy, have public health applications” [1]: it is difficult to know what counts as “modest” discrimination, and how much discrimination would have to improve to outweigh a given level of miscalibration. For instance, the discrimination estimated by Pfeiffer and colleagues as judged by the AUC would, to many, seem weak rather than modest.

The paper by Pfeiffer and colleagues is one of many current papers illustrating the need for quantitative evaluation of the clinical value of prediction models, needed to arm us to transfer rapidly growing medical knowledge to sound decision-making to the benefit of patients. Hopefully, we can have models and model evaluations that illuminate the whole spectrum, from public health decisions for groups of people to the vision of individualized medicine with individually tailored treatments.

## Abbreviations

AUC: area under the receiver operating characteristic curve

## References

[1] Pfeiffer RM, Park Y, Kreimer AR, Lacey JV Jr, Pee D, et al (2013) Risk prediction for breast, endometrial, and ovarian cancer in white women aged 50 y or older: derivation and validation from population-based cohort studies. PLoS Med 10: e1001492 doi:10.1371/journal. pmed.1001492 23935463 10.1371/journal.pmed.1001492 PMC3728034

[2] Schwartz LM, Woloshin S, Dvorin EL, Welch HG (2006) Ratio measures in leading medical journals: structured review of accessibility of underlying absolute risks. BMJ 333: 1248–1250. 17060338 10.1136/bmj.38985.564317.7C PMC1702463

[3] Ware JH (2006) The limitations of risk factors as prognostic tools. New Engl J Med 355: 2615–2616. 17182986 10.1056/NEJMp068249

[4] Pepe MS, Janes H, Longton G, Leisenring W, Newcomb P (2004) Limitations of the odds ratio in gauging the performance of a diagnostic, prognostic or screening marker. Am J Epidemiol 159: 882–890. 15105181 10.1093/aje/kwh101

[5] Mallett S, Haligan S, Thompson M, Collins GS, Altman DG (2012) Interpreting diagnostic accuracy studies for patient care. BMJ 344: e3999. 10.1136/bmj.e3999 22750423

[6] Fall K, Garmo H, Andrén O, Bill-Axelson A, Adolfsson J, et al (2007) Prostate-specific antigen levels as a predictor of lethal prostate cancer. J Natl Cancer Inst 99: 526–532. 17405997 10.1093/jnci/djk110

[7] Steyerberg EW, Vickers AJ, Cook NR, Gerds T, Gonen M, et al (2010) Assessing the performance of prediction models: a framework for some traditional and novel measures. Epidemiology 21: 128–138. 20010215 10.1097/EDE.0b013e3181c30fb2 PMC3575184

[8] Baker SG, Cook NR, Vickers A, Kramer BS (2009) Using relative utility curves to evaluate risk prediction. J R Soc Ser A Stat Soc 172: 729–748. 10.1111/j.1467-985X.2009.00592.x PMC2804257 20069131

[9] Pencina MJ, D'Agostino RB, Steyerberg EW (2011) Extension of net reclassification improvement calculations to measure usefulness of new biomarkers. Stat Med 30: 11–21. 21204120 10.1002/sim.4085 PMC3341973

[10] Vickers AJ, Elkin EB (2006) Decision curve analysis: a novel method for evaluating prediction models. Med Decis Making 26: 565–574. 17099194 10.1177/0272989X06295361 PMC2577036

[11] Cook NR (2007) Use and misuse of the receiver operating characteristic curve in risk prediction. Circulation 115: 928–935. 17309939 10.1161/CIRCULATIONAHA.106.672402

[12] Pencina MJ, D'Agostino RB Sr, D'Agostino RB Jr, Vasan RS (2008) Evaluating the added predictive ability of a new marker: from area under the ROC curve to reclassification and beyond. Stat Med 27: 157–172. 17569110 10.1002/sim.2929

[13] Pepe MS, Feng Z, Huang Y, Longton G, Prentice R, et al (2007) Integrating the predictiveness of a marker with its performance as a classifier. Am J Epidemiol 167: 362–368. 17982157 10.1093/aje/kwm305 PMC2939738

[14] Baker SG (2009) Putting risk prediction in perspective: relative utility curves. J Natl Cancer Inst 101: 1538–1542. 19843888 10.1093/jnci/djp353 PMC2778669

